# Research on Rural Landscape Spatial Information Recording and Protection Based on 3D Point Cloud Technology under the Background of Internet of Things

**DOI:** 10.1155/2022/3772108

**Published:** 2022-06-02

**Authors:** Li Yue, Zhang Yadong, Chen Jinjin

**Affiliations:** ^1^Faculty of Art and Design, Shandong Women's University, Jinan, Shandong 250300, China; ^2^Universiti Putra Malaysia, Faculty of Design and Architecture, Kuala Lumpur 43100, Malaysia

## Abstract

The village with historical and cultural accumulation not only is the witness of the interaction between human activities and natural environment but also contains a lot of intangible cultural heritage. It is an important research object in the history of human social development and has great reference value for the construction of modern Humayun settlements. With the continuous change of modern rural landscape, the traditional engineering drawing cannot meet the needs of people for spatial information measurement. There are some problems such as slow drawing, low precision, and poor benefit, so computer drawing must be used. Therefore, this paper puts forward the research on rural landscape spatial information recording and protection based on 3D point cloud technology under the background of Internet of Things and realizes the recording of rural landscape spatial information through the construction of 3D point cloud spatial information recording model. The experimental results show that the improved unit point cloud spatial information recording model has better feature extraction effect and feature point extraction efficiency, and can classify the point cloud more carefully. At the same time, the scene 3D point cloud classification method based on conditional random field can better deal with the interference factors in the outdoor landscape and show a better classification effect.

## 1. Introduction

The spatial integration model can effectively identify the spatial form of rural settlements. With the support of continuous data, we can not only dynamically observe the spatial evolution trend of rural settlements but also provide important support for rural land consolidation and rural planning. In terms of regional concept, the broad definition of village refers to the area that does not include cities or primitive uninhabited areas, and rural landscape refers to the food and other renewable natural resources obtained and produced from the natural environment through different farming and animal husbandry in rural areas [1]. In the development of human society, villages have always occupied an important position. Many villages with historical and cultural accumulation have been preserved through the baptism of time [2]. These villages relying on the natural environment not only retain many methods of land use, agricultural technology, and rural culture containing ecological wisdom. Moreover, it is of great value to the research on the history of human social development and also has great inspiration and reference value in the construction of modern human settlements [3]. Therefore, people have new views on the countryside and rural landscape, gradually realize the importance and value of rural landscape, and attribute rural landscape to the important task of heritage protection. However, the rural landscape is not invariable from beginning to end [4]. It has been in a state of continuous change and evolution, and most of the villages are still developing and changing [5]. At the same time, with the promotion of modern urban construction and new rural development, the social structure in the south of the ancient city of the county has changed, and the rural agricultural production is no longer the traditional farming method, but adopts modern agricultural technology [6]. In order to promote rural construction, many villages have developed corresponding rural tourism industry based on their own natural landscape and cultural landscape, which accelerates the change of rural landscape space [7]. From the perspective of rural landscape heritage protection, it is necessary to obtain the corresponding information from the rapidly changing rural landscape through the spatial information recording method of colleges and universities [8].

Spatial model is a methodology that can solve the spatial problems of rural landscape [9]. It refers to the specific spatial combination formed between different elements in rural landscape [10]. The spatial pattern of rural landscape is formed by the interaction between human activities and natural environment. Its development will be affected by factors such as productivity level, local culture, and natural environment [11]. The value of rural landscape heritage is reflected through the rural landscape spatial model [12]. Therefore, quantitative research on the rural spatial model is conducive to the authenticity, integrity, and diversity of rural landscape heritage protection [13]. Collecting and recording rural landscape spatial information through traditional surveying and mapping technology and spatial expression tools not only has high cost and long cycle time but also is difficult to deal with the rapidly changing rural landscape [14]. At the same time, the utilization rate of the information collected by traditional methods in three-dimensional spatial information is poor, the spatial feature expression effect of rural landscape is not good, and the accuracy of the measured data needs to be improved [15]. The development of point cloud technology has brought a new development direction for the recording and protection of rural landscape spatial information [16]. Point cloud technology includes a series of technologies such as the collection, processing, and visualization of point cloud data, which can vividly describe the three-dimensional coordinate information and reflectivity information of the sampling point data in the dense space of the measurement object and carry out three-dimensional models, lines, surfaces, and images of all kinds of large, complex, standard, or nonstandard entities and real scenes through computers' construction of various map data such as volume [17]. Compared with traditional surveying and mapping methods and spatial expression tools, it can obtain relevant information faster, cover a wider measurement area, collect data with higher accuracy, and have more intuitive expression. These advantages make point cloud technology more advantageous than traditional methods in rural landscape spatial information recording and protection. It can also provide better technical guarantee for the research of rural landscape spatial information recording and protection, and provide an important driving force for the development of rural landscape protection and spatial information [18].

Therefore, this paper puts forward the research on rural landscape spatial information recording and protection based on three-dimensional point cloud technology under the background of Internet of Things, and collects and processes the corresponding data of rural landscape by constructing the spatial information recording model of three-dimensional point cloud technology. This paper is mainly divided into three parts: the first part expounds the development and application of Sanyun point cloud technology; the second part is to build a rural landscape spatial information recording model based on three-dimensional point cloud technology, and improve the accuracy and reliability of rural landscape information collection and processing through the improvement of feature extraction technology and scene three-dimensional point cloud classification technology based on conditional random field; and the third part analyzes the application experimental results of rural landscape spatial information recording model based on three-dimensional point cloud technology.

This paper puts forward the research on rural landscape spatial information recording and protection based on 3D point cloud technology under the background of Internet of Things. The research innovation contributions include the following: the improved unit point cloud spatial information recording model has better feature extraction effect and feature point extraction efficiency, and can classify the point cloud more carefully. The scene 3D point cloud classification method based on conditional random field can better deal with the interference factors in the outdoor landscape, so as to show a better classification effect. In the application of rural landscape spatial information recording, 3D point cloud technology can complete the collection and recording of relevant spatial information in a short time, visualize the spatial information in a variety of ways, and understand the corresponding information more intuitively.

This paper is divided into four parts.  Section 1 expounds the research background of rural landscape spatial information based on three-dimensional point cloud technology under the background of Internet of things.  Section 2 expounds the citation of relevant literature and explains the wide application of 3D point cloud measurement technology.  Section 3 analyzes the construction of rural landscape spatial information recording model based on 3D point cloud technology.  Section 4 expounds the application experimental results of rural landscape spatial information recording model based on three-dimensional point cloud technology. Finally, the full text is summarized. The experimental results show that the improved unit point cloud spatial information recording model has better feature extraction effect and feature point extraction efficiency, and can classify the point cloud more carefully.

## 2. Related Work

**Figure 1:**
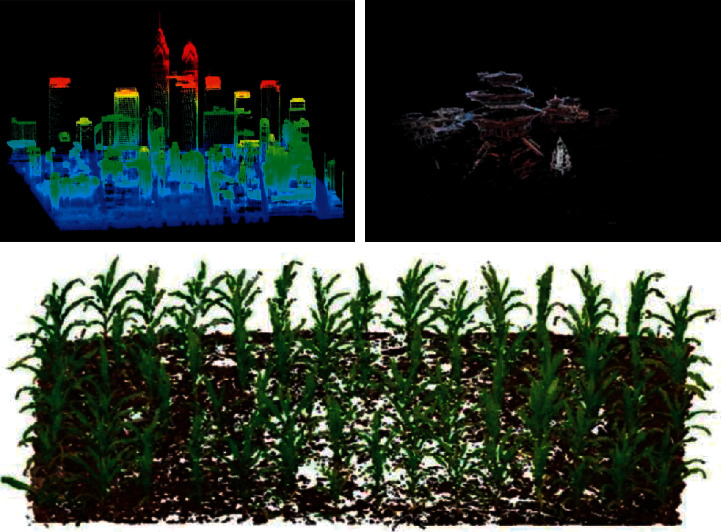
Spatial features and models extracted by 3D point cloud technology.

With the continuous development of scanning measurement technology and computer technology, 3D point cloud measurement technology has been paid more and more attention and applied in more and more fields. At the same time, the measurement of 3D point cloud technology has gradually developed from the original fixed single location measurement to motion measurement, and has been widely used in the fields of digital city, virtual vision, and so on [19]. According to the working principle of measuring equipment of 3D point cloud technology, its scanning technology can be divided into manual scanning technology and automatic scanning technology. The manual scanning technology can obtain the relevant information of the scanning object with arbitrary shape, and its accuracy is affected by the operation experience in the scanning process; automatic scanning can obtain relevant information data faster through three-dimensional scanner, which has the advantages of easy operation and automation, but it has certain requirements for the shape of the scanned object [20]. According to the contact between the equipment and the measured object in the data acquisition process of 3D point cloud technology, it can be divided into contact data acquisition method and noncontact data acquisition method. Among them, noncontact data acquisition method is widely used in cultural heritage protection. In the field of international cultural heritage, point cloud technology has become one of the important ways of spatial information recording of heritage sites, in which the representative technologies are lidar technology and digital close-range photogrammetry technology [21]. In China, some scholars have applied 3D point cloud technology to the protection of architectural cultural heritage and pointed out that it has five advantages. First, the noncontact data acquisition method of three-dimensional point cloud technology can reduce the damage to the heritage as much as possible and improve the accuracy of heritage ancient appearance restoration; second, 3D point cloud technology has fast data acquisition speed, which greatly improves the work efficiency, reduces the workload, and saves a lot of labor; third, compared with the traditional measurement methods, the data obtained by 3D point cloud technology have higher accuracy and reduce the accidental error of data; fourth, 3D point cloud technology changes the way of data measurement and improves the security of measurement; fifth, the data record of 3D point cloud technology is more detailed and comprehensive, and the visualization is diversified. Other scholars have recorded and protected the spatial data of Chinese classical garden heritage through three-dimensional point cloud technology, making the corresponding data more comprehensive and accurate [22]. In addition, with the advancement of agricultural modernization, some scholars have applied the three-dimensional point cloud technology to the measurement of farmland crop population growth parameters, which overcomes the limitations of traditional crop parameter measurement and realizes the nondestructive, efficient, and high-precision measurement of crop growth parameters [23]. As shown in Figure 1, the spatial features and models extracted by 3D point cloud technology.

3D point cloud technology obtains a large amount of point cloud data through the equipment and is vulnerable to the influence of the surrounding environment in the process of acquisition, which will have the problems of noise and hole. In order to ensure the accuracy and efficiency of the three-dimensional model of the object, it is necessary to preprocess before model construction. In addition, the lattice quality of point cloud data is the basis of its later classification, recognition, and feature extraction. In the actual environment, it will be affected by technical limitations and the surrounding environment, which will damage the integrity of the obtained point cloud data, and there are problems such as noise, interference points, occlusion in different point cloud areas, and uneven distribution of point cloud. At the same time, the spatial structure of point cloud is usually scattered, which improves the difficulty of defining the regional characteristics of different point clouds. Therefore, the segmentation and data processing of 3D point cloud has always been a research hotspot in this field. Some studies have proposed a 3D point cloud segmentation method based on set features, which strengthens the curvature expression ability through local weighted curvature feature description method and improves the efficiency and accuracy of edge feature point extraction [24]. In addition, the local partial differential equation is established based on the local finite element of the network to remove the anisotropic abnormal noise in the point cloud data. Some scholars proposed to introduce bilateral filtering in the denoising process to achieve the denoising effect, which has been widely used [25]. At present, there are several commonly used point cloud segmentation methods. The first is the segmentation based on edge characteristics, which determines the boundary data according to the geometric characteristics of point cloud. After determination, it will smoothly connect the point cloud boundary points, so as to obtain multiple point cloud subsets that do not want to intersect with each other and to achieve the effect of segmentation. Therefore, if it is necessary to segment point cloud data, the segmentation effect will be better when the edge characteristics are relatively prominent. The second is the clustering feature-based segmentation algorithm for attribute segmentation according to the feature vector of point cloud data. Generally, the clustering algorithms used include *k*-means algorithm, fuzzy clustering algorithm, and so on. This kind of algorithm is not easily affected by the spatial properties of point cloud in the process of segmentation and has a relatively stable segmentation effect. However, the most direct factor affecting the segmentation quality of clustering feature-based segmentation algorithm is the selection of point cloud spatial feature vector, and the point cloud spatial density will affect the setting of feature vector threshold to a great extent. The third is a model-based segmentation algorithm based on geometric mathematical model to achieve the purpose of point cloud segmentation through mathematical fitting. The algorithm can be constructed through a large number of mathematical formulas and has superior processing speed. At the same time, the fitting results obtained through the mathematical model will reduce the impact of local noise on the segmentation effect. However, before segmentation, the algorithm needs to classify different models, and then compare and classify the data and models, which means that the segmentation method needs a large number of object models, which is often used for rough segmentation of point cloud data. The fourth is a graph-based segmentation algorithm based on the disorder of point cloud data. The corresponding vertices in the data object graph of point cloud assign different weights that are not similar to the lines between different vertices according to the spatial characteristics, which will directly affect the segmentation results. The fifth is the region growth segmentation algorithm, which divides the point cloud data with similar attributes into independent regions with different attributes within the set threshold range. Different from the point cloud segmentation method based on edge information, the formation of seed points in the region growth segmentation method is based on a variety of spatial attributes of the point cloud and divided into different region subsets with large differences, but the differences in the same region are very small, so it has a more stable resistance to noise. At the same time, the segmentation quality of region growth segmentation method depends on the selection of seed points and the setting of growth criteria. Therefore, the abnormal points in point cloud data have relatively little impact on the segmentation results of point cloud, but their boundary delimitation is generally inaccurate. With the development of 3D point cloud technology, its processing, segmentation, feature extraction, and other technologies in point cloud data are also developing continuously, so as to improve the accuracy and reliability of 3D point cloud technology and provide better data services for the development of various fields.

## 3. Construction of Rural Landscape Spatial Information Recording Model Based on 3D Point Cloud Technology

Landscape model represents landscape design in the form of micro-entity. The landscape model is made up of the actual objects according to a certain proportion. It is an important tool and carrier for transmitting, explaining, and displaying design projects and design ideas. Therefore, in the model making and design, appropriate materials and processes should be selected according to the uses of different models, and esthetic principles and treatment technology should be considered. Because the landscape model shows the image of an intuitive entity in three-dimensional space, it is convenient for people to study the relationship between a landscape element and the environment. To make a feasible plan, the intuitiveness of the landscape model is also reflected in the integrity of the simulated landscape. It enables the viewer to evaluate and appreciate the complete spatial form and overall environment of the landscape through the model.

The three-dimensional point cloud technology realizes the collection, recording, and visualization of rural landscape spatial information mainly through four steps. First, it is necessary to sort out the landscape characteristics, spatial scope, constituent elements, and other types of information of the measured rural landscape, so as to provide corresponding basis for subsequent research. Second, appropriate methods, technologies, and equipment are used to collect the corresponding spatial information data for the space with different scales, different characteristics, and different accessibility. General rural landscape has diversified scales and rich elements, which need to be coordinated through a variety of equipment. Third, the rural landscape spatial model is constructed through image and point cloud processing software. Fourth, the spatial models of different rural landscapes need to be redeveloped according to different application requirements, such as the extraction of elevations and sections of spatial environment. It can be seen that the data collection of 3D point cloud technology is the basis of the construction, secondary development, and follow-up research of rural landscape spatial information model. The rural landscape is an outdoor landscape, and many factors in its surrounding environment have a certain impact on the data acquisition of 3D point cloud technology. Therefore, when building the feature extraction, segmentation, and classification model of 3D point cloud technology, it is necessary to improve the original 3D point cloud model algorithm, so as to achieve the target effect.

### 3.1. Feature Extraction and Segmentation Model Construction of 3D Point Cloud Technology

**Figure 2:**
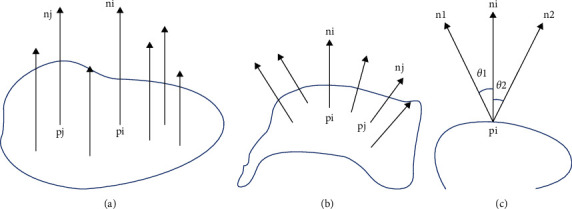
Schematic diagram of normal vector and angle between normal vectors in different surface areas. (a) Plane area. (b) Surface area. (c) Included angle of normal vector.

This paper adopts the feature point extraction method based on normal vector. The vector perpendicular to the local surface is the surface normal vector, which can describe the local surface direction, and the normal vector of any point on the surface is the normal vector. The idea of feature point extraction method based on normal vector is to solve the normal vectors of all data points contained in the point cloud data model and calculate the included angle between the normal vector of each data point and the normal direction of the corresponding adjacent points. The greater the included angle between the normal vectors, the greater the fluctuation between the corresponding data points and the adjacent point cloud data; that is, it has several sharp features; on the contrary, the smaller the angle between the normal vectors, the smoother the fluctuation between the point cloud data near the data points; that is, it has sparse geometric characteristics. As shown in Figure 2, the angle between normal vector and normal vector in different surface areas is shown. Based on the variation trend of the surface normal vector of the point cloud data, the purpose of selecting the appropriate feature points of the point cloud data can be realized by setting the appropriate threshold.

Let the coordinate of a certain point be *p*_*i*_, and its *k* adjacent point set be expressed as {*p*_1_, *p*_2_,…, *p*_*k*_}. Solve the mean value of all points, that is, solve its center of gravity *o*, as shown in the following formula:

(1) $$\begin{array}{c} o=\frac{1}{k}\sum_{i=1}^{k}p_{i}. \end{array}$$

The normal vector solution of the least squares fitting surface is to solve the minimum value of formula (2), as shown in the following formula:

(2) $$\begin{array}{c} f=\sum_{Nbh\;d\left(p_{i}\right)}\left\|\left(p_{i}–o\right)\cdot n\right\|. \end{array}$$

Furthermore, the problem of solving the minimum value of formula (2) is transformed into the problem of solving the minimum eigenvalue of covariance, as shown in the following formula:

(3) $$\begin{array}{c} \left[\begin{array}{ccc} \sum_{i}{\left(x_{i}-o_{x}\right)}^{2} & \sum_{i}\left(x_{i}-o_{x}\right)\left(y_{i}-o_{y}\right) & \sum_{i}\left(x_{i}-o_{x}\right)\left(z_{i}-o_{z}\right)\\ \sum_{i}\left(y_{i}-o_{y}\right)\left(x_{i}-o_{x}\right) & \sum_{i}{\left(y_{i}-o_{y}\right)}^{2} & \sum_{i}\left(x_{i}-o_{x}\right)\left(z_{i}-o_{z}\right)\\ \sum_{i}\left(z_{i}-o_{z}\right)\left(x_{i}-o_{x}\right) & \sum_{i}\left(z_{i}-o_{z}\right)\left(y_{i}-o_{y}\right) & \sum_{i}{\left(z_{i}-o_{z}\right)}^{2} \end{array}\right]. \end{array}$$

The coordinates of any point in the point cloud data are expressed as *x*_*i*_, *y*_*i*_, *z*_*i*_, and the corresponding center of gravity coordinates is expressed as *o*_*x*_, *o*_*y*_, *o*_*z*_. The minimum eigenvalue *λ*_0_ of the real symmetric matrix in formula (3) is solved, and the eigenvalue is the normal vector of the response point.

Unify the normal vectors with different directions obtained from different point cloud data points in one perspective, and take the change trend of the normal vector of a point, that is, the angle between the normal vector and the normal vector of the corresponding *k* adjacent point, to calculate the average value, as shown in the following formula:

(4) $$\begin{array}{c} f_{i}=\frac{1}{k}\sum_{j=1}^{k}\theta_{i}. \end{array}$$

The angle between the data point normal vector and its adjacent point normal vector is expressed as *θ*_*ij*_.

From the above formula, it can be seen that the size of neighborhood points has an impact on the calculation time of normal vector and the extraction effect of feature points. Therefore, the optimal neighborhood size is obtained by adding the neighborhood radius constraint method, so as to reduce the impact of noise on the calculation time of normal vector and improve the efficiency of feature point extraction. The neighborhood radius threshold is introduced and expressed as *r*. Each point selects the nearest *k* neighborhood points according to the radius threshold. The expression of the center point of each point is shown in the following formula:

(5) $$\begin{array}{c} c_{i}=\frac{1}{\left|k_{i}\right|}\sum_{p_{ij}\in N^{k_{i}}\left(p_{i}\right)}p_{ij}, \end{array}$$

where the center point is *c*_*i*_, ‖*p*_*ij*_‖ < *r*. Calculate the diffusion matrix and eigenvalue of the central point, select the reserved data points, and extract the feature points according to the size of the eigenvalue.

Set the point cloud set as *P*={*p*_*i*_}_*i*=1_^*N*^, where the nearest point in the neighborhood of each point is *N*^*k*_*i*_^(*p*_*i*_)={*p*_*ij*_},  *i* ≤ *j* ≤ *k*_*i*_, and the incidence matrix of each point is shown in the following formula:

(6) $$\begin{array}{c} C_{i}=\sum_{p_{ij}\in N^{k_{i}}\left(p_{i}\right)}\left(p_{ij}–c_{i}\right){\left(p_{ij}–c_{i}\right)}^{T}. \end{array}$$

The eigenvalue {*λ*_0_, *λ*_1_, *λ*_2_}, *λ*_0_ ≤ *λ*_1_ ≤ *λ*_2_ and the corresponding eigenvector {*e*_0_, *e*_1_, *e*_2_} of the incidence matrix are calculated. Retain the point set according to the set threshold, as shown in the following formula:

(7) $$\begin{array}{c} \frac{\lambda_{2}\left(p\right)}{\lambda_{1}\left(p\right)}<r_{12},\frac{\lambda_{3}\left(p\right)}{\lambda_{2}\left(p\right)}<r_{23}. \end{array}$$

The characteristic points of the remaining points are determined by the minimum characteristic value, as shown in the following formula:

(8) $$\begin{array}{c} \rho \left(p\right)\approx \lambda_{3}\left(p\right). \end{array}$$

The eigenvalue of the point is expressed as *ρ*(*p*).

In this paper, the method of 3D point cloud segmentation selects the region growth segmentation algorithm, which divides the point cloud data into independent regions with similar attributes according to a certain threshold range, and the attributes of different regions are different. The region growth segmentation algorithm has good robustness, and the algorithm principle is relatively simple and easy to operate. Its segmentation effect depends on the selection of seed nodes and the setting of growth criteria, and will be affected by the threshold setting. Therefore, the region growth segmentation algorithm is improved in the selection of seed nodes and the setting of growth criteria. The improved region growing method realizes region segmentation well and can segment several unconnected parts at the same time. We execute a cycle; that is, the program automatically determines a seed point and divides it. The number of seed points can be controlled by controlling the number of cycles. The program automatically determines the seed points, so that when the threshold and cycle times are determined, the image segmentation effect is certain, and the results can be reproduced. Let the seed node be the point with the smallest curvature, and estimate and sort the curvature of each point contained in the point cloud to be segmented. The average curvature *K*_*h*_ of a point in the surface is calculated as shown in the following formula:

(9) $$\begin{array}{c} 2K_{h}n=\lim\text{ diam}\left(A\right)⟶0\frac{\nabla A}{A}. \end{array}$$

The normal vector is expressed as *n*, an infinitesimal region around the point is expressed as *A*, the diameter of the region is expressed as diam(*A*), and ∇ is the gradient operator of the point. After discretization, the average curvature calculation formula is obtained, as shown in the following formula:

(10) $$\begin{array}{c} K_{h}\left(P_{i}\right)=\frac{1}{4A_{\min}}\sum j\in N\left(i\right)\left(\cos\;\alpha_{ij}+\cot\;\beta_{ij}\right)\left(P_{i}–P_{j}\right)\times n. \end{array}$$

The diagonals connecting edges *P*_*i*_ and *P*_*j*_ are *α*_*ij*_ and *β*_*ij*_, respectively.

### 3.2. 3D Point Cloud Classification of Scene Based on Conditional Random Field

Rural landscape belongs to outdoor scene, and the traditional outdoor point cloud classification method is lack of estimation of 3D point cloud data characteristics of large scale, high complexity, and strong noise outdoor scene. Therefore, this paper selects the scene 3D point cloud classification method based on conditional random field. Each point in the constructed conditional random field based on point cloud represents a node, the random variable is represented as *L*={*L*_1_, .., *L*_*N*_}, its range is *L*_*i*_ ∈ {*l*_1_,…, *l*_*k*_}, and each point classifies a label and satisfies the scoring function. The point cloud classification task is equivalent to a simple learning problem. The discrimination model to be constructed in the learning process can be proposed from the outdoor scene probability model *P*_*w*_(*l|x*). In this model, the dependence between features is expressed by the potential function of the log linear model of the associated Markov model, as shown in formula (11), which is the potential function of a single node

(11) $$\begin{array}{c} \log\;\varphi_{i}\left(l_{k}\right)=\omega_{n}^{k}\cdot x_{i}. \end{array}$$

When the node label is assigned *k*, the feature weight is expressed as *ω*_*n*_^*k*^, and the feature vector of three-dimensional space points is expressed as *x*_*i*_. The similarity of the two connected nodes is expressed by the edge model, and the correlation is expressed by the edge potential function, as shown in the following formula:

(12) $$\begin{array}{c} \log\;\varphi_{ij}\left(l_{k},l_{k}\right)=\omega_{e}^{k}\cdot x_{ij}. \end{array}$$

The two connected nodes are assigned the weight value of the same label, which is expressed as *ω*_*e*_^*k*^ and ∀*l*_*k*_ ≠ *l*_*o*_, log  *φ*_*ij*_(*l*_*k*_, *l*_*o*_)=0. Finally, through the logarithmic model of joint conditional probability, as shown in the following formula:

(13) $$\begin{array}{c} \log\;P_{w}\left(y|x\right)=\sum_{i=1}^{N}\sum_{k=1}^{K}\left(\omega_{n}^{k}\cdot x_{i}\right)y_{i}^{k}+\sum_{ij\in E}\sum_{k=1}^{K}\left(\omega_{e}^{k}\cdot x_{ij}\right)y_{i}^{k}y_{j}^{k}-\log\;Z_{w}\left(x\right). \end{array}$$

The *Z*_*w*_(*x*) calculation formula is as follows:

(14) $$\begin{array}{c} Z_{w}\left(x\right)=\sum_{{}_{y}^{'}}\prod_{i=1}^{N}\varphi_{i}\left(y_{i}^{\prime }\right)\prod_{ij\in E}\varphi_{ij}\left(y_{i}^{\prime },y_{j}^{\prime }\right). \end{array}$$

Let the row vector be *w*={*w*_*n*_, *w*_*e*_}, *w*_*n*_={*w*_*n*_^1^,…, *w*_*n*_^*k*^}, *an*  *d* *w*_*e*_={*w*_*e*_^1^,…, *w*_*e*_^*k*^} and its length be *K*(*d*_*n*_+*d*_*e*_). Redefine *y* as the column vector *y*={*y*_*n*_, *y*_*e*_}^*T*^, *y*_*n*_={…, *y*_*i*_^1^,…, *y*_*i*_^*k*^,…}, *an*  *d* *y*_*e*_={…, *y*_*ij*_^1^,…, *y*_*ij*_^*k*^,…}, and its length is *K*(*N*+|*E*|), and then, the size of matrix *X* is expressed as *K*(*d*_*n*_+*d*_*e*_) × *K*(*N*+|*E*|), and finally, formula (13) is transformed into the following formula:

(15) $$\begin{array}{c} \log\;P_{w}\left(y|x\right)=wXy-\log\;Z_{w}\left(x\right). \end{array}$$

## 4. Application Experiment Results of Rural Landscape Spatial Information Recording Model Based on Three-Dimensional Point Cloud Technology

**Figure 3:**
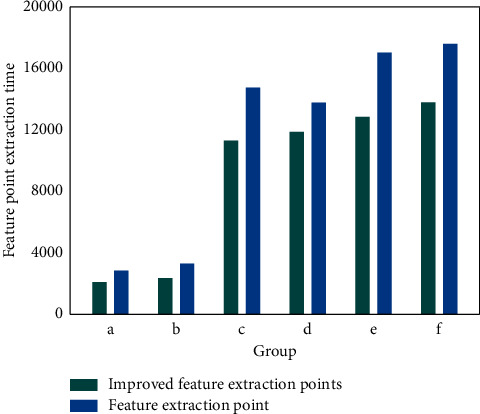
Comparison between the improved feature extraction algorithm and the original feature extraction algorithm in feature point extraction results.

This paper selects a rural landscape for the application experiment of rural landscape spatial information recording model based on three-dimensional point cloud technology, and randomly selects six different rural landscapes for the experiment of feature extraction points. As shown in Figure 3, the improved feature extraction point algorithm is compared with the original feature extraction algorithm in feature point extraction results. As can be seen from the figure, compared with the original feature extraction algorithm, the improved feature extraction algorithm integrating the neighborhood constraint method has significantly improved the feature extraction effect, and the feature extraction time has been greatly reduced. This shows that the improved feature extraction algorithm integrated with neighborhood constraint method can better extract the required data points, greatly improve the extraction efficiency on the basis of improving the extraction effect, and show good application performance in time application.

**Figure 4:**
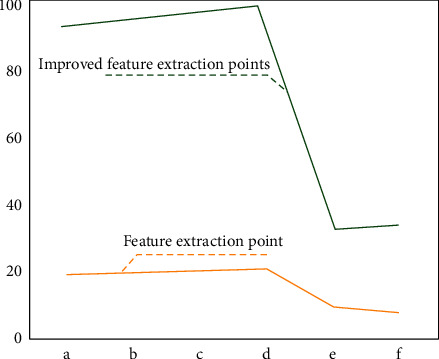
The comparison results of the number of point cloud clusters obtained after segmentation between the improved region growth algorithm and the traditional region growth algorithm.

This paper selects three small-scale rural landscapes for the point cloud data segmentation effect test, as shown in Figure 4, which is the comparison result of the number of point cloud clusters obtained after the segmentation of the improved region growth algorithm and the traditional region growth algorithm. It can be seen from the data in the figure that compared with the traditional region growth algorithm, the improved region growth algorithm can better segment the image, separate the nonplanar region, and maintain the good boundary. This shows that the improved region growth algorithm can segment more carefully, the quasidetermination of segmentation is better, the performance is more stable, and it can overcome the problem of local region over segmentation.

**Figure 5:**
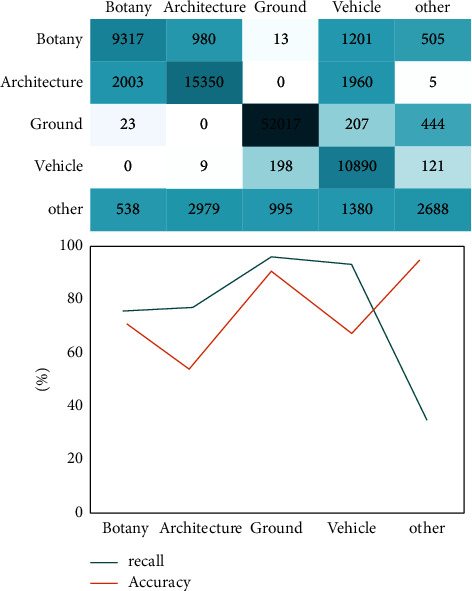
Experimental results of rural landscape point cloud classification based on random forest.

**Figure 6:**
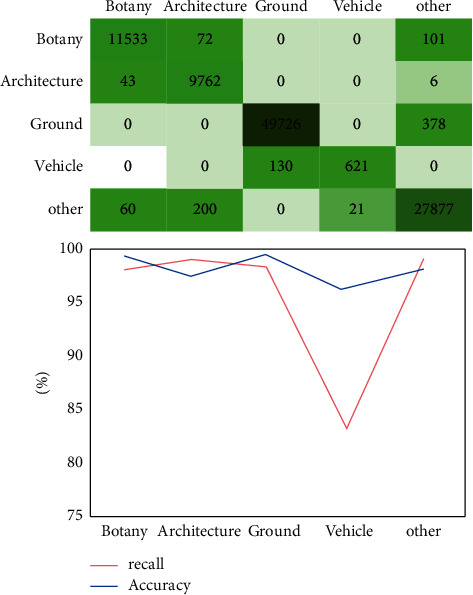
Experimental results of rural landscape point cloud classification based on conditional random field scene.

This paper selects the outdoor scene classification method based on random forest and the scene 3D point cloud classification method based on conditional random field for experimental comparison. As shown in Figures 5 and 6, the experimental results of rural landscape point cloud classification based on random forest and rural landscape point cloud classification based on conditional random field scene are shown, respectively. Random forest is a local classifier related to decision tree, which is composed of many decision trees. When classifying the 3D point cloud of outdoor scene, it is necessary to add category labels to the points contained in each 3D space. Then, the final classification label is selected by voting from the decision tree. Therefore, the outdoor scene classification method based on random forest mainly obtains the classification results according to the characteristics of a single point cloud, which will have some errors due to the voting method. As can be seen from the results in the figure, in terms of recall rate, the classification results of rural landscape point cloud based on conditional random field scene are higher than those of outdoor scene based on random forest. This shows that the scene 3D point cloud classification method based on conditional random field not only considers the local area features of a single point but also takes into account the similarity between adjacent point features and the relationship between points and adjacent areas. The outdoor scene classification based on random forest only infers according to the shape feature description of three-dimensional points. Therefore, the scene 3D point cloud classification method based on conditional random field has the advantages that the outdoor scene classification based on random forest does not have, and shows a better effect in point cloud classification.

**Figure 7:**
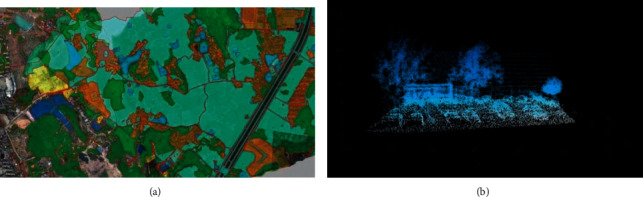
Spatial information collection and visualization of a rural landscape obtained by 3D point cloud technology.

As shown in Figure 7, it is the spatial information collection and visualization of a rural landscape obtained through three-dimensional point cloud technology. It can be seen from figure (a) that the three-dimensional point cloud technology can classify and divide the rural landscape in detail according to different attribute characteristics, and record the corresponding spatial information, so as to provide a data basis for further analysis. (b) This figure is the visualization of the laser point cloud model of houses and trees in the countryside, which can intuitively see the profile of buildings and trees and the corresponding centimeter accuracy.

Experiments show that the three-dimensional point cloud technology can quickly collect, quantitatively measure, and analyze the spatial information of different scales in different areas of rural landscape, and express it in a variety of ways. Compared with the traditional surveying and mapping method, it has a great improvement in measurement accuracy and efficiency. At the same time, the visualization of the spatial model of the rural landscape through the three-dimensional point cloud model saves the cost of manual modeling, and there is no contact between the measured objects in the whole process of data collection, which greatly improves the protection of the relatively fragile elements in the rural landscape.

## 5. Conclusion

There is a close relationship between the development of human society and rural landscape. Rural landscape is the result of the interaction between human activities and natural environment. It is not only an important carrier of human history and culture, but also its retained intangible cultural heritage, land methods, and agricultural technology have certain research and reference value for human settlements and agricultural development. With the rise of environmental awareness, people pay more and more attention to the protection and research of rural landscape. However, the rural landscape has been changing. The traditional measurement methods have responded to such changes and cannot meet people's needs for rural landscape measurement. With the development of science and technology and information technology, 3D point cloud technology can quickly complete the collection and recording of spatial information without contact. Therefore, this paper puts forward the research on the recording and protection of rural landscape spatial information based on 3D point cloud technology under the background of Internet of Things and records the spatial information of rural landscape through Internet of Things technology and 3D point cloud technology. At the same time, rural landscape belongs to outdoor landscape, and there are many factors that affect the data acquisition of 3D point cloud technology. This paper improves the feature extraction and region growth segmentation algorithm of 3D point cloud. The experimental results show that the improved 3D point cloud feature extraction effect is better, the extraction time is shorter, the point cloud classification can be carried out more carefully, the nonplanar regions can be separated, and the boundary is good. The three-dimensional point cloud classification method based on conditional random field has more classification advantages than the outdoor scene classification based on random forest and can achieve better classification effect. In addition, in the application of rural landscape spatial information recording, three-dimensional point cloud technology can complete the collection and recording of relevant spatial information in a short time, visualize the spatial information in a variety of ways, and understand the corresponding information more intuitively. However, there are still some problems to be solved in this paper. The improved three-dimensional point cloud technology still has a great deficiency, which cannot realize threshold adaptation, and the threshold needs to be given manually. The next step is to start from here and find a solution to realize the program to automatically determine the threshold and obtain better results.

## Data Availability

The data used to support the findings of this study are available from the corresponding author upon request.
